# In Silico Approaches: A Way to Unveil Novel Therapeutic Drugs for Cervical Cancer Management

**DOI:** 10.3390/ph14080741

**Published:** 2021-07-29

**Authors:** Diana Gomes, Samuel Silvestre, Ana Paula Duarte, Aldo Venuti, Christiane P. Soares, Luís Passarinha, Ângela Sousa

**Affiliations:** 1 CICS-UBI—Health Sciences Research Centre, Universidade da Beira Interior, 6201-506 Covilhã, Portugal; dianarouca@gmail.com (D.G.); sms@ubi.pt (S.S.); apcd@ubi.pt (A.P.D.); 2 Associate Laboratory i4HB—Institute for Health and Bioeconomy, Faculdade de Ciências e Tecnologia, Universidade NOVA, 2819-516 Caparica, Portugal; 3 UCIBIO—Applied Molecular Biosciences Unit, Departamento de Química/Departamento Ciências da Vida, Faculdade de Ciências e Tecnologia, Universidade NOVA de Lisboa, 2829-516 Caparica, Portugal; 4 Laboratório de Fármaco-Toxicologia-UBIMedical, Universidade da Beira Interior, 6200-284 Covilhã, Portugal; 5 CNC—Center for Neuroscience and Cell Biology, University of Coimbra, 3004-504 Coimbra, Portugal; 6 C4—Cloud Computing Competence Centre, UBIMedical, Universidade da Beira Interior, Estrada Municipal 506, 6200-284 Covilhã, Portugal; 7 HPV-UNIT-UOSD Tumor Immunology and Immunotherapy, IRCCS Regina Elena National Cancer Institute, 00144 Rome, Italy; aldo.venuti@ifo.gov.it; 8 Department of Clinical Analysis, School of Pharmaceutical Sciences, São Paulo State University (UNESP), Campus Ville, Araraquara 14800-903, São Paulo, Brazil; soarescp@fcfar.unesp.br

**Keywords:** cervical cancer management, computer-aided drug design, E6 inhibitors, in silico studies, human papillomavirus

## Abstract

Cervical cancer (CC) is the fourth most common pathology in women worldwide and presents a high impact in developing countries due to limited financial resources as well as difficulties in monitoring and access to health services. Human papillomavirus (HPV) is the leading cause of CC, and despite the approval of prophylactic vaccines, there is no effective treatment for patients with pre-existing infections or HPV-induced carcinomas. High-risk (HR) HPV E6 and E7 oncoproteins are considered biomarkers in CC progression. Since the E6 structure was resolved, it has been one of the most studied targets to develop novel and specific therapeutics to treat/manage CC. Therefore, several small molecules (plant-derived or synthetic compounds) have been reported as blockers/inhibitors of E6 oncoprotein action, and computational-aided methods have been of high relevance in their discovery and development. In silico approaches have become a powerful tool for reducing the time and cost of the drug development process. Thus, this review will depict small molecules that are already being explored as HR HPV E6 protein blockers and in silico approaches to the design of novel therapeutics for managing CC. Besides, future perspectives in CC therapy will be briefly discussed.

## 1. Introduction

Cancers are some of the deadliest pathologies, and according to Globocan, the global cancer burden in 2020 increased to 19.3 million cases and 10 million cancer deaths. With these new data, it is estimated that 1 in 5 people will develop cancer during their lifetime, and 1 in 8 men and 1 in 11 women will die from the disease [1]. Thereby, as logical consequence, novel pharmacotherapy approaches have increased in the recent years [2]. In particular, cervical cancer (CC) is considered the fourth cause of death among women worldwide with its establishment being associated with human papillomavirus (HPV) infection [3]. Considering the data available by Globocan regarding this pathology, there were 341,831 deaths in 2020 with a higher incidence in low-income countries (LIC). In fact, in less developed regions such as Africa, Asia, and South America, CC is the primary cancer found in women, which can be due to the lack of screening programs, limited resources and access to health care, or even anti-vaccination movements [4,5]. In developed countries, the incidence of CC cases is lower due to better health services and the high availability of HPV prophylactic vaccines, which constitutes a great step in the prevention of HPV-associated cancers. However, the prophylactic vaccines have only been effective when administered in healthy patients, and they are not able to exert a therapeutic effect or treat an established infection [6].

The current treatments for CC are based on excisional or ablative procedures, surgical resection, radiotherapy, or chemotherapy, which do not specifically target the oncogenic properties of HPV, and therefore lesion recurrence can occur [7]. In addition, most of these procedures can affect normal tissues and can have potential side effects, including bleeding, which cause patient discomfort and can reduce life quality [5]. These constraints highlight the need to improve the current therapeutic approaches by combining strategies or proposing new compounds to offer more specific and less invasive treatments, without affecting healthy tissues. Hence, the scientific community has been focused on different ways to combat CC. A strategy with great potential consists of finding new anticancer agents by targeting the major oncoproteins responsible for HPV-driven carcinogenesis, E6 and E7. In fact, the discovery of the E6 protein X-ray crystal structure, available in protein data bank (PDB ID: 4GIZ and 4XR8, accessed on 20th May 2021), led to an increase in the use of in silico approaches to uncover potential E6 inhibitors [8].

Drug discovery and development is a very expensive and time-consuming process, which can take 10 to 15 years until a drug reaches the market. In the last decades, the pharmaceutical industry has been employing computer-aided drug design (CADD) techniques to accelerate drug development, intending to reduce time, costs, and failures, namely in the final stage [9,10]. This analysis is based on calculated properties and prediction models for drug therapeutic targets and identification of safety liabilities. Typically, CADD can be divided into three categories: structure-based, ligand-based, and hybrid methods [11,12]. The structure-based approaches, including docking and the application of molecular dynamics simulations, use the 3D structure of the target molecule to screen potential ligands. These methods evaluate ligand recognition by the target molecule and the prediction and characterization of binding sites as well as binding affinity [9,12]. For instance, molecular docking is one of the most applied techniques to select promising molecules from large libraries by predicting the orientation of a compound towards the target and characterizing ligand–target interactions. Molecular dynamics techniques entail the motion principles to molecules and are frequently used to perform binding mode studies and to predict the stability of a ligand–target complex, giving a deeper understanding to the researchers on the interaction of a ligand to a biomolecular target [9,11,12,13]. On the other hand, ligand-based methods, including similarity searching, pharmacophore modeling, and quantitative structure–activity relationship (QSAR) studies, use the information of groups of small molecules with different structures capable of interacting with the target to identify new and powerful compounds [12,14]. These methods are usually applied when the 3D structure of the target is not available and assume that analogous compounds show similar biological activity and interaction with the target. QSAR modeling allows understanding the effects of structural variables on biological activity to develop compounds with enhanced and optimal pharmacological profiles. Another ligand-based method is similarity searching, which is mostly applied in filtering compounds from big libraries based on the assumption that compounds with structural similarity can have similar bioactivity [9]. When the 3D structure of the target is available, as well as the ligands’ structure, it is possible to use hybrid methods. This means combining structure-based and ligand-based methods to perform some types of pharmacophore modeling or to predict the activity considering the biological profile of tested compounds against several targets [12]. In fact, combining CADD methods can be more effective once their advantages complement one another [11,12]. Given the importance of pharmacokinetics and toxicity properties of selected compounds, in silico ADMET (Absorption, Distribution, Metabolism, Excretion, and Toxicity) filters can also be applied to eliminate compounds with potential undesirable physiological qualities [9]. With the improvement of technology and bioinformatic tools, the use of in silico approaches for drug development, mainly in preliminary studies, has increased over the years.

Hence, in this review, we intend to summarize the current treatment used for CC stages induced by HPV persistent infection, present the small molecules that are already being explored as inhibitors/blockers for E6 protein, and retrospectively analyze the studies published in the last years that applied in silico approaches to the design of novel therapeutics for CC treatment. In addition, we will concisely discuss the future perspectives for CC management.

## 2. HPV, CC Development, and Clinical Treatment

HPV is the etiological agent associated with CC. There are about 200 HPV genotypes of high-risk (HR HPV) and low-risk (LR HPV) identified. HPV16 and 18 are responsible for more than 70% of invasive CC. The HPV genome presents tropism for epithelial cells, and the infections appear mainly in the anogenital tract [3,5,15] and head and neck anatomic sites. While HPV is the hallmark of CC development [15], the classic major risk factors to head and neck cancer are tobacco and alcohol, but in the past few decades, human papillomavirus (HPV) has emerged as a novel risk factor [16,17]. The biology and life cycle of human papillomavirus have been reviewed elsewhere [18]. However, we considered it helpful to readers to give a brief insight into the HPV life cycle that may lead to carcinogenesis. 

The HPV genome encodes eight genes; the L1 and L2 structural proteins constitute the capsid that protects the viral genome, and the E1, E2, E4, E5, E6, and E7 proteins are involved in replication, transcription regulation, and oncogenesis. E6 and E7 oncoprotein expression disrupt the cell repair mechanisms by degradation or inhibition of the tumor suppressor proteins p53 and retinoblastoma protein (pRB), respectively, resulting in the immortalization and cellular transformation of infected cells [19,20,21]. The viral life cycle begins with viral particles arriving in the basal layer of the squamous epithelia via micro-abrasions. After reaching the nucleus the viral DNA is replicated, and low quantities of E1, E2, E6, and E7 proteins are produced early in the infection, halting normal keratinocyte development. Then, E2 recruits E1 to increase the number of viral episome copies, which continue to rise as the epithelium differentiates. E6 and E7 proteins are abundantly expressed in the top differentiating epithelial layers, resulting in uncontrolled cell proliferation, and the viral life cycle is completed when L1 and L2 proteins are expressed in the epithelium’s highest layer. As a result, the viral genome is encapsulated, and mature virions are released [22,23]. Although many women contract HPV, most infections are eliminated or controlled by the immune system after 1–2 years [24]. The establishment of persistent infection is associated with the appearance of cervical lesions, since the accumulation of DNA damage caused by HR HPV E6 and E7 interactions with tumor suppressors, p53 and pRb, causes apoptosis suppression and uncontrolled proliferation. The chronically infected cells lead to the establishment of cervical intraepithelial neoplasia (CIN), as CIN grade I, which over time can evolve to CIN grade II, III, or invasive carcinoma [23,24].

CC is staged according to different systems. The most widely used is the FIGO system proposed by the International Federation of Gynecology and Obstetrics in collaboration with the International Union Against Cancer (IUCC) [7]. Table 1 summarizes the different stages of FIGO’s system with the current treatment for managing CC. 

All surgical interventions can cause side effects or complications, including bleeding and damage in the tissue nearby, and simple or radical hysterectomy results in infertility or bladder/bowel dysfunction [25]. With the spread of cancer to other tissues (metastasis), surgery is no longer a viable possibility. Radiation therapy (RT) uses radiation to destroy cancer cells, and it is possible to affect mainly the zones with the tumor in the lower abdomen in CC cases. Some side effects can be infertility, discomfort, and menopause. Chemotherapy involves the administration of cytotoxic drugs to interfere with cell proliferation, killing rapidly dividing cells. The currently approved drugs by the FDA for CC treatment include, among others, bleomycin sulfate, topotecan, pembrolizumab, bevacizumab, cisplatin, paclitaxel, vinorelbine, ifosfamide, fluorouracil, and gemcitabine [26]. Nevertheless, these treatments are unable to effectively distinguish healthy from cancer cells, affecting several types of dividing cells throughout the entire body. This effect can result, namely, in a higher risk of anemia, bleeding, and infections [3,25]. Hence, the design and development of therapeutic agents that are efficient, non-toxic, and capable of distinguishing cancerous from healthy tissue are essential. In addition, for managing early-stage CC, the employment of less invasive methods to improve quality of life and decrease treatment-related sexual dysfunction is a necessity.

## 3. E6 Protein

As mentioned before, E6 and E7 proteins of HR HPV play a major role in CC development once its growth is dependent on sustained E6/E7 expression. The interest in HR HPV E6 protein as a potential therapeutic target has arisen in the past years. This protein is essentially found in the cell nucleus and is constituted by 150–160 amino acids and two zinc-binding motifs. In 2013, Zanier and co-workers published in PDB the 3D structure of HPV16 E6 protein bound to the E6AP interaction domain (ID: 4GIZ) [27]. More recently, the same research group also published the 3D structure of the E6-E6AP-p53 trimeric complex (ID: 4XR8) [28]. The wild-type (wt) HPV16 E6 protein forms intermolecular disulphide bonds, which makes it very difficult, if not impossible, to purify [29]. Thus, Zanier and colleagues used an E6 nonfull-sequence structure (4C/4S mutant) to achieve its crystallization, where four cysteines were replaced with four serine residues. The E6 4C/4S mutant maintains the ability to degrade p53 in a very similar way to that of HPV16 wt E6 [30]. These crystallographic findings determine the identification and design of novel compounds to block the HR HPV E6 oncoprotein action with higher efficiency. As the E7 protein 3D structure is still unknown/resolved, this explains why researchers are mainly directing their efforts in developing strategies to block E6 to the detriment of E7.

The E6 protein binds to E6AP (E6-associated protein), a ubiquitin ligase required for the interaction with p53 protein. The formation of the trimeric E6-E6AP-p53 complex is responsible for the ubiquitination and degradation of p53, resulting in p53-dependent apoptosis blockage. The crystallization data revealed that HPV16 E6 is composed of an N-terminal and a C-terminal connected by an alpha helix, forming the hydrophobic binding pocket for E6AP [27]. E6 targets other structures that are involved in several molecular pathways, bringing down some cancer hallmarks. For example, HR HPV E6 protein is able to activate telomerase by transcriptional up-regulation of TERT (reverse transcriptase component of telomerase). There are two mechanisms proposed for this phenomenon, and both require the E6-E6AP complex formation. Briefly, one of the models proposes that the E6-E6AP complex can bind to NFX1-91 (repressor of TERT transcription) causing its degradation and releasing the transcriptional repression at the TERT promoter. On the other model, the E6-E6AP complex binds to c-myc, which may displace the inhibitory USF transcriptional repressor from the E box in the TERT promoter. Other mechanisms are possible once the telomerase activation is not well understood. Besides telomere elongation, TERT can participate in apoptosis inhibition and allow cell proliferation, which increases the probability of malignant conversion [31]. Besides E6AP binding, E6 only from high-risk HPV can bind to the PDZ-domain family of proteins through its C-terminal, although this region is not necessary for p53 interaction [28,32,33]. Thus, HR HPV E6 protein complexes, along with the different proteins that bind to E6, affect several biological functions such as cell survival, DNA damage, and cell cycle progression. Considering the problems associated with current treatments for CC and the important role of E6 oncoprotein in the progression of HR HPV infection towards cervical lesions, the inhibition/blockage of its function could be a promising and specific therapeutic strategy. Indeed, several authors have shown that decreased HR HPV E6 expression by RNA interference, flavonoids, and intracellular antibodies could restore p53 levels and induce apoptosis in HPV-positive cell lines [34,35]. Hence, a presentation of the advances made in anticancer drug design and development for the HR HPV E6 protein using computational methods will be performed. For that, three databases (PubMed, Web of Science, and ScienceDirect) were used to search articles published after 2015 with the following keywords: E6 inhibitors AND in silico; E6 inhibitors AND molecular docking; E6 protein AND in silico; E6 protein AND molecular docking.

### 3.1. E6-E6AP Complex Inhibitors

**Figure 1:**
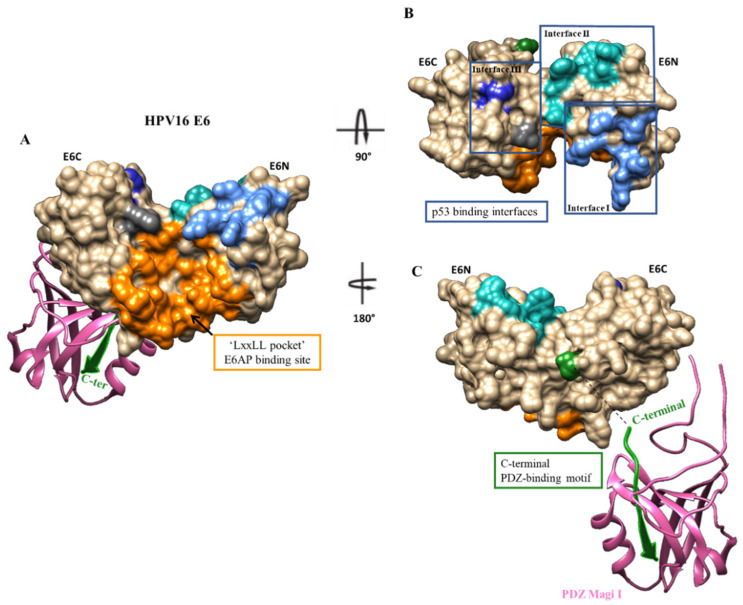
A map of different binding sites of the HPV16 E6 protein using PDB ID 4XR8 and 2KPL on Chimera software. Surface representation of E6 residues that participate in the binding with E6AP (**A**), p53 (**B**), and the PDZ domain (**C**). **A**—Orange residues participate in E6AP binding; **B**—The three interfaces of contact between E6 and p53 are colored in light, medium, and dark blue. **C**—The C-terminal PDZ binding motif is represented in green. E6N: N-terminal; E6C: C-terminal.

The HR HPV E6 protein has different binding pockets, including the hydrophobic pocket where the LxxLL motif of E6AP binds, the PDZ-binding domain, and the p53 binding site, Figure 1 [27,28]. Until recently, the most explored area to achieve inhibition has been the hydrophobic pocket, to the detriment of the HR HPV E6PDZ domain. 

Researchers have been taking advantage of the E6/E6AP complex 3D structure available on PDB (ID: 4GIZ) to understand the interactions involved between both proteins and to apply in silico methods to design potential inhibitors. E6AP is required to induce E6-mediated p53 degradation by inducing structural changes in the E6 protein, which allow the formation of the trimeric complex E6/E6AP/p53 [28]. The hydrophobic pocket able to recognize the LxxLL motif is mainly constituted by the following amino acid residues: Leu100, Leu50, Cys51, Val53, Val62, Leu67, Tyr32, Tyr70, and Ile73 [27].

#### 3.1.1. Synthetic Compounds

**Figure 2:**
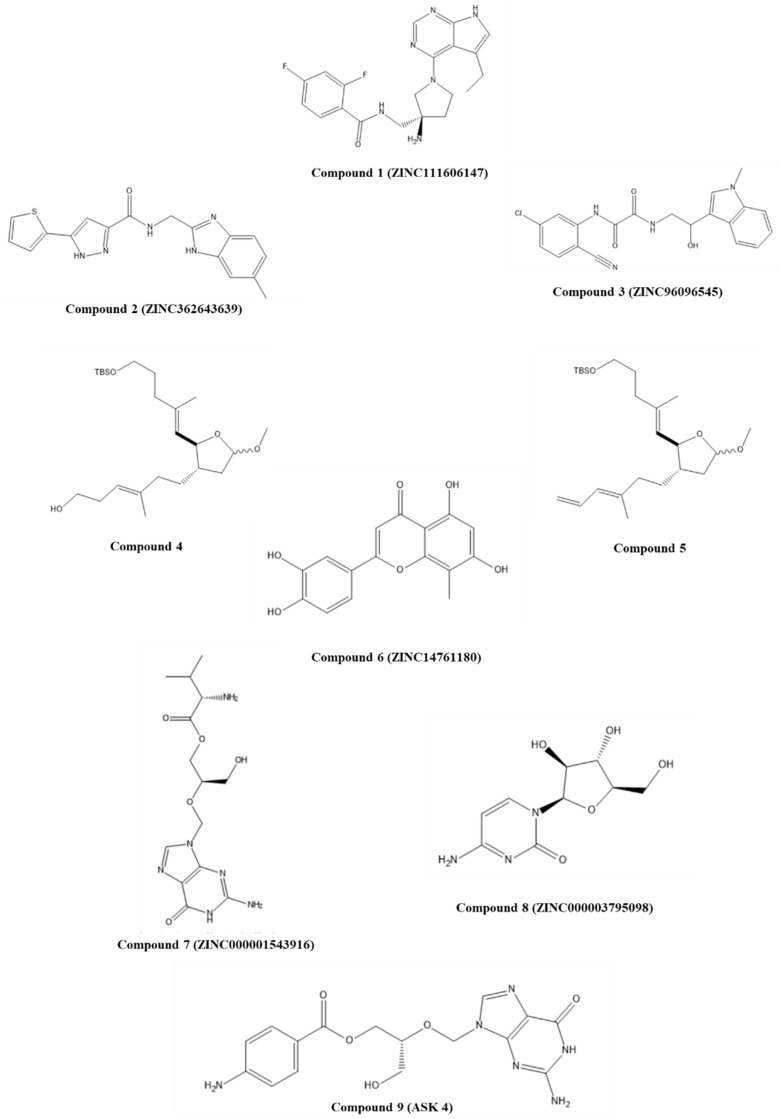
Structures of studied synthetic compounds as E6/E6AP inhibitors. IUPAC name of compounds—compound **1**: (*S*)-*N*-((3-amino-1-(5-ethyl-7*H*-pyrrolo [2,3-*d*]pyrimidin-4-yl)pyrrolidin-3-yl)methyl)-2,4-difluorobenzamide; compound **2**: *N*-((6-methyl-1*H*-benzo[*d*]imidazol-2-yl)methyl)-5-(thiophen-2-yl)-1*H*-pyrazole-3-carboxamide; compound **3**: *N*^1^-(5-chloro-2-cyanophenyl)-*N*^2^-(2-hydroxy-2-(1-methyl-1*H*-indol-3-yl)ethyl)oxalamide; compound **4**: (*E*)-6-((2*S*,3*S*)-2-((*E*)-5-((*tert*-butyldimethylsilyl)oxy)-2-methylpent-1-en-1-yl)-5-methoxytetrahydrofuran-3-yl)-4-methylhex-3-en-1-ol; compound **5**: *tert*-butyl(((*E*)-5-((2*S*,3*S*)-5-methoxy-3-((*E*)-3-methylhexa-3,5-dien-1-yl)tetrahydrofuran-2-yl)-4-methylpent-4-en-1-yl)oxy)dimethylsilane; compound **6**: 2-(3,4-dihydroxyphenyl)-5,7-dihydroxy-8-methyl-4*H*-chromen-4-one; compound **7**: (*S*)-(*R*)-2-((2-amino-6-oxo-1*H*-purin-9(6*H*)-yl)methoxy)-3-hydroxypropyl 2-amino-3-methylbutanoate; compound **8**: 4-amino-1-((2*R*,3*S*,4*S*,5*R*)-3,4-dihydroxy-5-(hydroxymethyl)tetrahydrofuran-2-yl)pyrimidin-2(1*H*)-one; compound **9**: (*R*)-2-((2-amino-6-oxo-1*H*-purin-9(6*H*)-yl)methoxy)-3-hydroxypropyl 4-aminobenzoate.

Ricci-López and co-workers developed an in silico pipeline to propose potential inhibitors of E6/E6AP interaction [36]. For this, the HPV16 E6 sequence (UniProt ID: P03126) and the template retrieved from the ternary complex E6/E6AP/p53 (PDB ID: 4XR8) were used to obtain the full-length structure of the HPV-16 E6 protein by homology modeling once the E6 protein was crystallized by using a 4C/4S mutant, as mentioned earlier. Twenty-six compounds described in the literature as capable of binding to E6 were used as reference molecules and queries in the Zinc15 database to build a compound library with almost 35,000 molecules using a structural similarity approach. ZINC15 is a public access database provided by the Irwin and Schoichet Laboratories in the Department of Pharmaceutical Chemistry at the University of California, San Francisco (UCSF). It contains over 230 million purchasable compounds in ready-to-dock, 3D formats and over 750 million purchasable compounds, and it is possible to search for analogues. It is widely used for virtual screening, ligand discovery, and pharmacophore screens, among others. Compounds were then filtered considering ADME properties, and those with the highest potential were submitted to molecular docking studies considering the hydrophobic pocket as the binding site. After the binding free energy calculations of the best-docked complexes, compound **1** (ZINC111606147), compound **2** (ZINC362643639), and compound **3** (ZINC96096545) (Figure 2) were selected for further evaluation through molecular dynamics simulations. When compared to luteolin (reference ligand), these three compounds had higher affinity to the E6 protein, and all docked complexes were stable and capable of inhibiting E6/E6AP interaction [36].

Senthilkumar and colleagues described ansiomelic acid (AA), a compound isolated from the plant *Anisomeles malabarica*, as being able to inhibit E6 and E7 protein expression and induce apoptosis in HPV-positive cancer cells. Then, they decided to perform a structure–activity relationship study with several AA analogues to identify potent inhibitors of HPV E6 and E7 oncoproteins [37]. Molecular docking was used to predict the affinity of 26 AA-derived compounds towards the hydrophobic pocket of the E6 protein (PDB ID: 4GIZ) using AA as control. Of these, compounds **4** and **5** (Figure 2) showed the highest docking scores −65.74 Kcal/mol and −63.66 Kcal/mol, respectively, and lower IC_50_ values (HPV-positive cell lines, SiHa and HeLa) were observed when compared to AA and other compounds. In addition, they displayed less toxicity towards fibroblasts than commercial drugs and were able to inhibit p53 degradation mediated by the E6 protein, according to the in vitro studies performed [37].

Rietz and his team followed a different approach. For this, small-molecule probes, which can modulate protein interactions, were used to evaluate the recognition features of the E6 protein by means of binding and functional assays [38]. In this context, authors intended to understand the contribution of the substituents in positions 2 and 6 on the benzopyranone scaffold explored and to determine which E6 features are involved in binding. Interestingly, it was observed that charged groups in position 6 and non-polar substituents in position 2 displayed higher activity. Molecular dynamics simulations with analogs of these small molecules and exploring a set of mutations in different amino acid residues allowed the authors to conclude that a group of arginines (Arg10, Arg55, Arg102, Arg129, and Arg131) play a major role in the shape of the E6 helical binding groove, as well as in molecular recognition of the binding partners. Therefore, these last results can be of relevance for structure-based targeting of HPV E6 [38].

Another strategy was developed by Kumar and colleagues using ligand-based and structure-based methods [39]. They built an e-pharmacophore model for virtual screening based on the amino acid residues involved in the interaction of E6 (ID: 4GIZ) and a peptide reported by Zanier and co-workers [35]. The ligands were selected based on literature information, and 2-aminobenzothiazole and the luteolin chromone moiety were used as query molecules to found potential compounds on the ZINC 15 database. Then, 6000 compounds were screened using the pharmacophore model developed, and molecular docking studies predicted the best compounds based on dock scores and interactions with amino acid residues of the binding site. In addition, ADME analysis and molecular dynamics simulations were performed to find the best hit. The chromone derivative, compound **6**, ZINC14761180 (Figure 2) showed the most relevant interactions, and molecular dynamics of the complex ZINC14761180-E6 protein evidenced good stability in the binding pocket [39]. These findings can be a starting point for further design and synthesis of new E6 inhibitors. Following this work, the authors decided to explore a different approach but with the same objective [40]. For this, a drug repurposing approach based on the FDA-approved drugs library was applied. This methodology consists of identifying a different therapeutic use for an available or approved drug. Approved drugs have acceptable ADMET properties; therefore, this is an effective method as it involves less time, lower costs, and reduces the probability of an undesirable ADMET profile in clinical trials [10]. After the compounds’ screening through a pharmacophore model, docking, molecular dynamics simulations, and ADME analysis, the selected hits with the highest potential were compound **7** (ZINC000001543916—valganciclovir; anti-viral drug) and compound **8** (ZINC000003795098—cytarabine; anticancer drug) (Figure 2). Both drugs are purine or pyrimidine nucleoside analogues, which showed that these scaffolds can be applied to design novel E6 inhibitors. Interestingly, compound **9**, ASK4 (Figure 2), which is a valganciclovir derivative, also showed promising results. Molecular dynamics simulations indicated that these ligands could form a stable complex with E6 protein, and it was also evidenced that they have an acceptable ADME profile [40].

**Figure 3:**
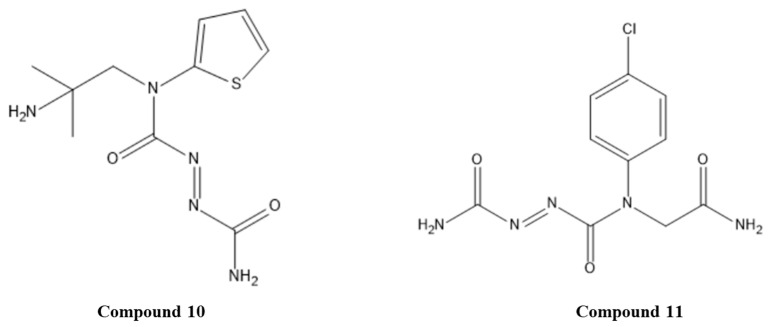
Structures of studied synthetic compounds as E6/E6AP inhibitors acting by ejecting zinc ions. IUPAC name of compounds—compound **10**: (*E*)-*N*^1^-(2-amino-2-methylpropyl)-*N*^1^-(thiophen-2-yl)diazene-1,2-dicarboxamide; compound **11**: (E)-*N*^1^-(2-amino-2-oxoethyl)-*N*^1^-(4-chlorophenyl)diazene-1,2-dicarboxamide.

The zinc-finger motif was proposed as strictly necessary for E6 protein function. Therefore, mutations in zinc-fingers interfere with E6/E6AP complex formation and cellular transformation. Concerning these data, Choudhury and co-workers selected specific disulfide (C13, C14, C16, R2, R15, R19) and azoic (C4) compounds, as controls based on their ability to eject zinc from the zinc finger motif of E6 protein and inhibit E6/E6AP interaction, proved by in vitro studies. Then, derivatives of these compounds were generated, and their ADME properties were predicted to exclude undesired compounds. Molecular docking, using the 3D structure of the C-terminal zinc-binding domain of the E6 protein (PDB ID: 2FK4), was performed for the selected ligands. Afterward, ligands with the highest binding score, such as compound **10**, *(E)-N*-(2-amino-2-methylpropyl)-*N*-(thiophen-2-yl)diazene-1,2-dicarboxamide, and compound **11**, (*E*)-*N*-(2-amino-2-oxoethyl)-*N*-(4-chlorophenyl)diazene-1,2-dicarboxamide, both azoic derivatives (Figure 3), were used for pharmacophore modeling (ligand-based method). The predicted amino acid residues involved in the interaction with the ligands are Tyr15, Trp55, Asn50, Leu23, Leu33, Ser5, Ile24, Ile51, Arg47, Arg54, Arg25, Lys45, Lys38, Phe48, Cys26, and Ala1. Some of these residues are found to be conserved among HPV6, HPV11, HPV16, and HPV18 zinc fingers. This demonstrates the possibility of the zinc finger domain to be a drug target [41]. 

#### 3.1.2. Natural Compounds

**Figure 4:**
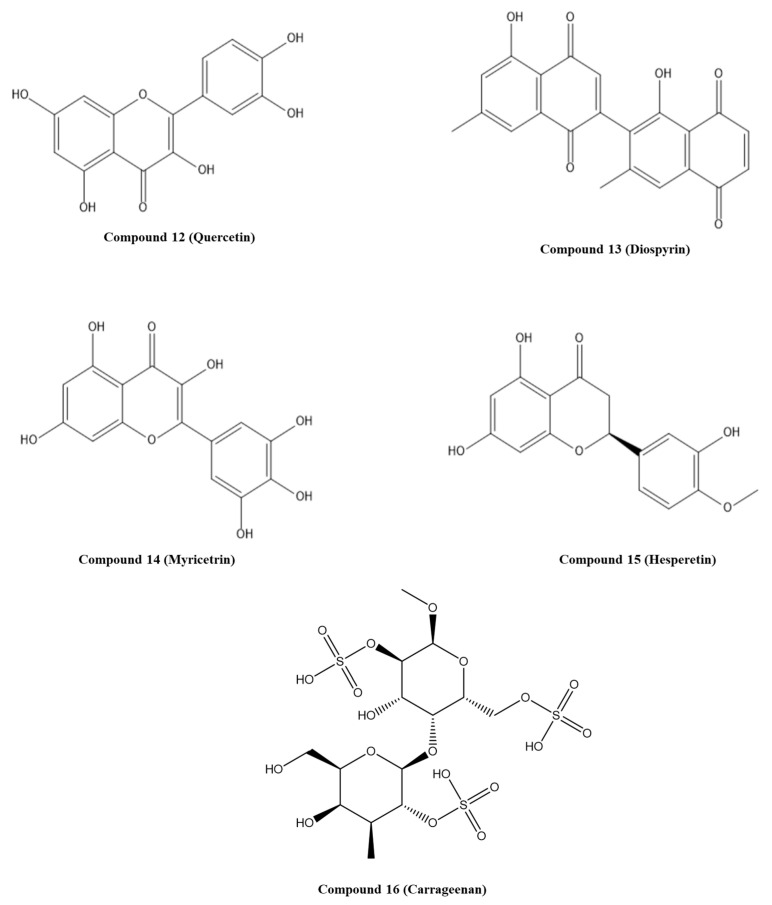
Structures of the studied natural compounds as E6/E6AP inhibitors. IUPAC name of compounds—compound **12**: 2-(3,4-dihydroxyphenyl)-3,5,7-trihydroxy-4*H*-chromen-4-one; compound **13**: 1’,5-dihydroxy-3’,7-dimethyl-[2,2’-binaphthalene]-1,4,5’,8’-tetraone; compound **14**: (*S*)-5,7-dihydroxy-2-(3-hydroxy-4-methoxyphenyl)chroman-4-one; compound **15**: (*S*)-5,7-dihydroxy-2-(3-hydroxy-4-methoxyphenyl)chroman-4-one; compound **16**: ((2*R*,3*R*,4*S*,5*R*,6*S*)-4-hydroxy-3-(((2*S*,3*R*,4*S*,5*R*,6*R*)-5-hydroxy-6-(hydroxymethyl)-4-methyl-3-(sulfooxy)tetrahydro-2*H*-pyran-2-yl)oxy)-6-methoxy-5-(sulfooxy)tetrahydro-2*H*-pyran-2-yl)methyl hydrogen sulfate.

Plant-derived compounds have been used in the prevention and treatment of different clinical conditions, and several of these natural products possess beneficial effects against several types of cancers, including cervical, colon, skin, breast, and prostate cancers [42]. In addition, many natural products are abundantly available and therefore can constitute a cost-effective way to obtain active pharmaceutical ingredients, including for cancer treatment [43]. Therefore, some researchers have focused on investigating the potential of natural compounds as E6 inhibitors. Clemente-Soto and his research team studied the ability of quercetin, compound **12** (Figure 4), a flavonol belonging to the flavonoid group, to inhibit p53 degradation mediated by E6 through in silico and in vitro studies [44]. According to molecular docking results, quercetin was able to bind in three different sites of E6 (ID: 4GIZ). Interestingly, it was predicted that the lowest energy (−7.08 Kcal/mol) and interactions with crucial amino acids for E6-E6AP formation (such as L50, L100, R102, R131) occurred in site II (corresponds to the hydrophobic pocket). As reference ligands, CAF24, C170, and luteolin were used because it was previously demonstrated that they were able to bind the E6 protein and disrupt the E6AP association [34]. Additionally, in vitro studies demonstrated that quercetin was capable to reactivate p53 and induce G2 phase cell cycle arrest and apoptosis in HPV-positive cancer cell lines [44].

Kumar’s research group took advantage of both 3D structures of the E6 protein available on PDB (ID: 4GIZ and 4XR8) to perform a protein–protein alignment and identify the amino acid residues that suffer conformational changes when the E6/E6AP complex recruits p53 [45]. The residues Arg8, Tyr32, Cys51, Tyr70, Ser74, and Arg131 demonstrated conformational changes when the E6/E6AP complex bound to the p53 protein. Then, an e-pharmacophore model was built based on the amino acid residues present in the predicted cavity and screened against 27,354 compounds. Molecular docking and molecular dynamics approaches were used to study the hits previously selected, where compound **13**, diospyrin (Figure 4), was identified as the best hit. H-bond interactions with Tyr32, Cys51, Ser74, and Arg131 amino acid residues were predicted, which might be important for an inhibitor to interact with the E6 protein [45].

Kolluru and his research team stated that E6 can inhibit the apoptotic pathway by binding to adaptor molecule FADD (Fas-associated death domain); therefore, the inhibition of this interaction could be a promising strategy for CC treatment [46]. As there is no information about the FADD binding site on the E6 protein, the authors decided to identify the flavonol binding pocket on HPV 16 E6 (ID: 4GIZ) protein by studying six flavonols reported in the literature as E6 inhibitors. First, a blind docking was performed to identify different pockets, followed by the determination of amino acid residues involved in interactions with ligands. Amino acids Cys51, Leu50, Arg102, Arg131, Leu67, Val62, and Gln107 were the most common among binding pockets that have scores correlated with the previously described IC_50_ (determined by the interaction of GST-E6 and His-caspase 8), indicating their importance in E6 inhibition. Compound **14**, myricetin (Figure 4), demonstrated the highest docking score in most of the binding pockets, which agrees with the IC_50_ reported in the literature. The efficacy of these ligands may be increased by conjugation with multivalent glycocalixarenes, which are known to interact with biological macromolecules [46].

Other researchers, such as Prakash and his team, studied the anticancer effect of compound **15**, hesperetin (Figure 4), a plant-isolated flavonoid, on CC via in vitro and molecular docking studies [47]. First, a preliminary study of hesperetin’s effective cytotoxicity towards HeLa cells was performed. Then, molecular docking with crystal structure PDB ID: 4XR8 was applied to understand the binding mode and interaction of the hesperetin–E6 protein complex. The binding energy score was −5.58 Kcal/mol, and different bonds were established with the active site of the E6 protein, specifically H-bonds with Trp132 and Asp98, carbon–hydrogen bond with Arg102 and Leu100, and hydrophobic pi-alkyl interaction with Arg102, Arg131, and Leu100. These results demonstrated that hesperetin can bind in the hydrophobic pocket of E6 and hinder the interaction of E6 with p53 [47].

Kamma and co-workers focused on ligands available on natural sources, including carrageenan, curcumin, and papain, to target the E6 protein (PDB ID: 6SIV) [48]. Molecular docking revealed minimal binding energy (−10.7 Kcal/mol) for compound **16**, carrageenan, being considered the best ligand of this group. However, this is a preliminary study, and it is fundamental to perform in vitro studies to confirm the carrageenan behavior towards CC and non-cancer cells.

Although plant-derived compounds present many advantages as anticancer agents, their usual low availability on tumor sites could represent a drawback. Thus, their administration using adequate drug delivery systems or combination with approved drugs could be a promising way to improve their bioavailability and allow their delivery to target cells, including for CC management [43,49].

The activation of interferon regulatory factor 3 (IRF3) is dependent on the kinase binding site within the autoinhibitory domain (AD), which is blocked by E6 protein [50]. The interaction of E6 with E6AP prevents apoptosis after p53 degradation causing cell cycle disruption. Both IRF3 and E6AP E6 have specific leucine-rich motifs. In the IRF3 case, it is the N-terminal that participates in E6 binding, where for the E6AP it is the C-terminal. Therefore, HPV escape from the antiviral response is suggested to be possible through the interaction of E6 protein with IRF3 [50]. However, the mechanism of IRF3 inactivation by E6 is not completely understood. Thus, Shah and colleagues used in silico approaches to explore this mechanism [51]. The N-terminal of IRF3 comprises two leucine-rich clusters that are assumed as E6-specific binding motifs. The binding affinity of these motifs towards the E6 protein was evaluated through protein–protein docking and molecular dynamics simulations using the E6/E6AP complex structure (PDB ID:4GIZ), as a control to corroborate the protocol described by the authors. After investigation of IRF3 stable residues and identification of E6 residues with high binding energy, the binding mode of E6 inhibitors reported in the literature was explored. Molecular docking with 20 ligands (natural and synthetic compounds) into the hydrophobic pocket of E6 was performed, and their binding affinities and behaviors were evaluated through computational mutagenesis and drug resistance scanning. Computational mutagenesis was applied to study the stability of each ligand-bound E6 complex, where the polar arginine residues of the E6 pocket were mutated into alanine residues. Then, the drug resistance of E6 was determined considering the difference of binding affinities of using wild-type or mutant residues. It was noticed that the stability was compromised when Arg131 was mutated into alanine, whereas Arg102 Ala mutation reduced the ligand-binding affinities. The change in the binding affinities suggests that E6 might become more resistant to drugs when Arg131 and Arg102 are mutated into neutral amino acid residues. The data obtained indicated that the LxxLL motifs of IRF3 bind within the hydrophobic pocket of E6 and the polar areas (Arg55, Arg102, and Arg131), significantly affecting the stability of the LxxLL-E6 complex. Despite the new findings where the E6 binding to IRF3 might inhibit the kinase-mediated protein activation, the fact that the polar patches are inconsistent among HR HPV species may compromise an unsuccessful treatment due to point mutation that could make drugs ineffective [51].

### 3.2. E6-p53 Complex Inhibitors

CC satisfies the criteria of “oncogene addiction”, which means that tumor cell development occurs due to the activity of one or some genes [52]. As E6 is the main protein responsible for p53 degradation, several researchers recently focused their attention on designing and developing potential inhibitors of E6/p53 binding by employing in silico methods. In fact, the direct disruption of the p53 binding site to E6 can be useful in the development and optimization of specific inhibitors, improving the likelihood of preventing off-target effects. 

#### 3.2.1. Natural Compounds

**Figure 5:**
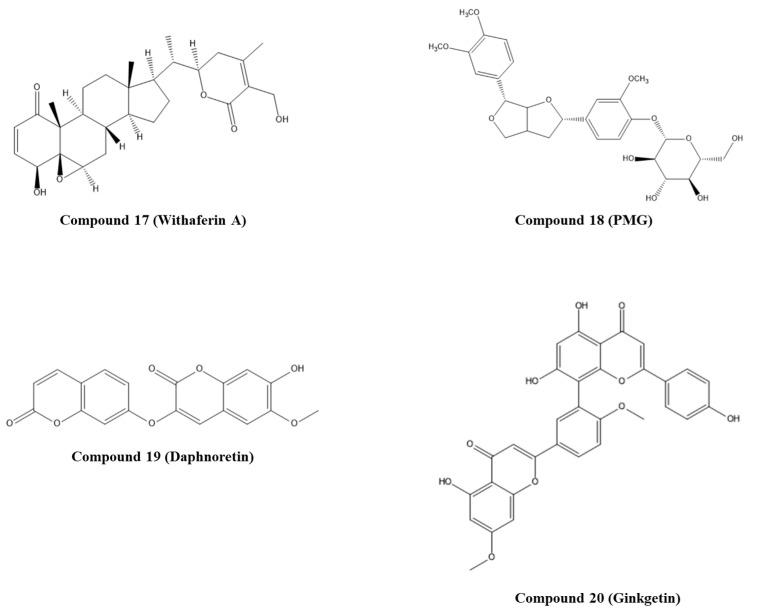
Structures of the studied natural compounds as E6/p53 inhibitors. IUPAC name of compounds—compound **17**: (4*S*,4a*R*,5a*R*,6a*S*,6b*S*,9*R*,9a*S*,11a*S*,11b*R*)-4-hydroxy-9-((*S*)-1-((*R*)-5-(hydroxymethyl)-4-methyl-6-oxo-3,6-dihydro-2*H*-pyran-2-yl)ethyl)-9a,11b-dimethyl-5a,6,6a,6b,7,8,9,9a,10,11,11a,11b-dodecahydrocyclopenta[1,2]phenanthro[8a,9-b]oxiren-1(4*H*)-one; compound **18**: (2*S*,3*R*,4*S*,5*S*,6*R*)-2-(4-((2*S*,6*R*)-6-(3,4-dimethoxyphenyl)hexahydrofuro[3,4-*b*]furan-2-yl)-2-methoxyphenoxy)-6-(hydroxymethyl)tetrahydro-2*H*-pyran-3,4,5-triol; compound **19**: 7-hydroxy-6-methoxy-3-((2-oxo-2*H*-chromen-7-yl)oxy)-2*H*-chromen-2-one; compound **20**: 5,7-dihydroxy-8-(5-(5-hydroxy-7-methoxy-4-oxo-4*H*-chromen-2-yl)-2-methoxyphenyl)-2-(4-hydroxyphenyl)-4*H*-chromen-4-one.

Kumar and co-workers explored natural compounds described in the literature as able to block HPV infection towards the E6 protein of HPV18 [53]. The 3D structure of E6 HPV18 was obtained by homology modeling using the amino acid sequence available in NCBI (GenBank ID: NP_040310.1), and the E6 HPV16 protein (PDB ID: 4GIZ) was employed as a structure template. After structure validation, molecular docking with 12 ligands was performed to elucidate the interactions with E6 HPV18. The binding site of p53 to E6 HPV18 was revealed by sequence comparison with E6 HPV16 and set on the residues 108–117 (CQKPLNPAEK). All studied compounds interacted with the p53 binding site on the E6 protein. However, the lowest binding energy (−5.85 Kcal/mol) was predicted for compound **17**, withaferin A (Figure 5) interacting through H-bonds with four amino acid residues of E6 (Glu116, Asn113, Asn122, and Ser140) [53]. Following this work, Atabaki and colleagues performed in silico and in vitro studies to evaluate the behavior of phytochemicals of *Jurinea macrocephala* subsp. *Elbursensis* in CC cell lines and their interaction with E6 HPV18 [54]. From this plant, three compounds were isolated, namely, 4-hydroxypectorolide-14-*O*-acetate, 4-hydroxy pectorolide, and pinoresinol monomethyl ether-*β*-D-glucoside (PMG), and their toxicity toward cancer cells was investigated. Of these, compound **18**, PMG (Figure 5), displayed significant toxicity on HeLa cells; therefore, molecular docking was performed to understand if it could be a potential E6 inhibitor. Using doxorubicin as control, the PMG showed higher binding energy (−4.75 Kcal/mol) and interacted through H-bonds with three amino acid residues (Arg119, Leu112, and Tyr99) [54].

Mamgmain and co-workers explored five natural compounds—colchicine, curcumin, daphnoretin, ellipticine, and epigallocatechin-3-gallate—as potential E6 inhibitors [55]. The 3D structure of the E6 protein was built through homology modeling and the crystal structure PDB ID: 4GIZ as a template. The binding sites predicted were chosen for molecular docking studies. The highest binding affinity, −8.3 Kcal/mol, was attributed to compound **19**, daphnoretin (Figure 5), which interacts through H-bonds with E6 amino acid residues Tyr39, Cys58, Ser78, Gln114, and Arg138 [55].

Considering the different molecules/proteins (E6AP, p53, and c-Myc) that bind to E6 protein and can interfere with normal cell function, Nabati and co-workers investigated more than 100 already described plant-derived compounds with anticancer and antiviral properties for the different protein binding sites [56]. These compounds were filtered based on their ADMET properties, and twenty compounds were selected for molecular docking studies. The crystal structure of the E6 protein PDB ID: 4GIZ was used. Compound **20**, ginkgetin (GK, Figure 5), extracted from *Gingko biloba* leaves, was the most effective in binding to all sites (E6AP, p53, and Myc) on the E6 protein, and the lowest binding energy was determined. In particular, it was predicted that GK forms five H-bonds with Arg55, Cys51, Val53, and Tyr60 on the E6AP binding site; on the p53 binding site it should form H-bonds with the same amino acid residues of the E6AP binding site; on Myc binding site, it can interact with Pro5 and Arg8 by forming four H-bonds. Considering the GK bioactivities described in the literature, including antitumor and antiviral properties, and ability to induce apoptosis in cancer cells, this study could be a starting point for the development of potential E6 inhibitor in vitro studies [56].

With the crystallization of the ternary complex E6/E6AP/p53 by Zanier and co-workers in 2016, it was possible to achieve a better structural understanding of the amino acid (aa) residues that participate in the binding interface of E6-p53. Actually, the aa residues described in the literature before 2016, or used as the binding site of p53 to E6 in some of the studies mentioned above, were predicted by computational methods since there was no information about the real binding site [28,57,58]. Even now, some authors chose to use computational methods for this purpose, while other authors used the information of the p53 binding site discovered in 2016. According to this study, the E6AP C-terminal does not substantially contribute to contacts with p53, and the E6/p53 interface can be divided into three sub-interfaces. In sub-interface I the E6 residues Glu7 and Glu18 establish interaction with p53 residues, where mutations in Glu18 impair the ternary complex formation and p53 degradation. Likewise, the hydrogen bond formed through Gln104 and Gly105 of p53 to Arg8 and Gln6 of E6 alters the conformation of the E6 N-terminal. Mutagenesis in the amino acid residues Phe2, Pro5, Arg8, or Pro9 prevented the ternary complex formation and p53 degradation. Sub-interface II has the aa residues that mediate vital contacts to p53 and are important for p53 degradation. The residues consist of Phe47, Asp44, and Asp49, which correspond to the most conserved positions in HR HPV. In addition, Ile23, His24, and Tyr43 provide hydrophobic contacts with p53. Finally, sub-interface III involves hydrophobic interactions between Leu114 and Trp146 of p53 and Leu100 and Pro112 of the E6 C-terminal [28].

#### 3.2.2. Synthetic Compounds

**Figure 6:**
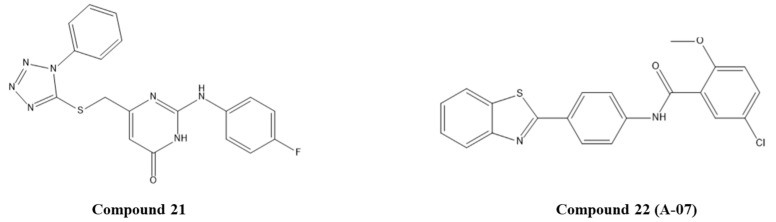
Structures of the studied synthetic compounds as E6/p53 inhibitors. IUPAC name of compounds—compound **21**: 2-((4-fluorophenyl)amino)-6-(((1-phenyl-1*H*-tetrazol-5-yl)thio)methyl)pyrimidin-4(3*H*)-one; compound **22**: *N*-(4-(benzo[*d*]thiazol-2-yl)phenyl)-5-chloro-2-methoxybenzamide.

Celegato and colleagues used the crystal structure of the E6/E6AP/p53 complex (PDB ID: 4XR8) to perform an in silico screening of small-molecule libraries against the central region of the p53-binding cleft of E6 [58]. This region was chosen due to its significant role in p53 binding and degradation as well as the high conservation among HR HPVs. A structure-based screening of three databases of commercially compounds was performed using chain F of the complex, and a filter step was applied considering the interactions with the residues Asp49 and Phe47 of E6. Twenty-nine compounds were selected and tested through their ability to rescue p53 by in vitro studies. This strategy allowed the selection of three compounds, but only pyrimidinone 21 (Figure 6) affected the viability of CC cells without affecting healthy cells. It was also found that this compound could re-establish p53 intracellular levels and transcriptional activity, reduce the viability and proliferation of HPV-positive cancer cells, and block 3D cervospheres formation [58]. Molecular dynamics simulations with E6-compound 20 complex were performed to gain further insight into the binding mode of compound **20**. The complex showed stability by the formation of a double H-bond with Asp49 and by hydrophobic interactions with Phe47 and established other interactions with Leu12, Cys16, and Ile23. This study suggested that compound **21** can be a starting point for the development of specific anti-HPV drugs.

Modi and co-workers explored the anticancer properties of 10 benzothiazole derivatives in CC cell lines. Later, by in silico and in vitro studies, they aimed to identify the molecular pathways involved in the apoptosis induced by *N*-(4-(benzo[d]thiazol-2-yl)phenyl)-5-chloro-2-methoxybenzamide (compound **22** or A-07, Figure 6) [59]. To perform in silico analysis in this context, the E6 and p53 crystal structures were obtained from PDB (ID: 4GIZ and 4XR8, respectively). Then, A-07 was evaluated by molecular docking against each protein where the active site was identified by the metaPocket server. It was predicted that this benzothiazolyl derivative was able to interact with both proteins, specifically with 10 amino acids of E6 and 14 amino acids of p53. These results suggest that A-07 (Figure 6) can hinder ternary complex formation and prevent p53-mediated intrinsic apoptosis in SiHa cells, as demonstrated by in vitro studies [59].

The natural and synthetic compounds presented here as HR HPV E6 protein inhibitors by either target E6/E6AP complex or the E6/E6AP/p53 complex formation are a valid starting point for drug design and development in cervical cancer management. However, strategies that combine both targets, the hydrophobic pocket and the p53-binding cleft of E6 protein, could result in an effective way to disrupt the E6/E6AP/p53 complex to specifically affect the viability of HPV-positive cancer cell lines and proposing specific anti-HPV therapies.

## 4. Future Perspectives

CC management still needs improvements, as current therapies are mainly surgery, chemotherapy, or radiotherapy. Several drugs have been proposed for treating patients with CC, but most do not overcome clinical trials due to low efficacy [36]. Bearing in mind the role of HR HPV E6 protein in the development and progression of HPV infection to cervical lesion or even invasive carcinoma, the inhibition of E6 function could be useful for treating CC. Results reported in this review support the idea that combining in silico approaches and in vitro studies could lead to a rise in the number of molecules understudy to block/inhibit E6 protein. According to Franconi and co-workers and Duenas-Gonzalez and co-workers, there are no clinical studies using natural or synthetic compounds as E6 or E7 inhibitors yet [43,60], due to low affinity and/or potency on in vitro and in vivo studies. Thus, the employment of in silico methodologies in the drug development process can be a great help to quickly find potential inhibitors and circumvent possible undesirable properties of compounds. Therefore, identification and functional evaluation of proteins associated with E6 could provide an insight into CC carcinogenesis and thus allow the design of specific strategies towards tumor cells. Moreover, it is essential to search and find cost-effective treatment options that could bring better outcomes for the patients. A strategy in this context could explore the potential use of plant-derived compounds, usually associated with lower toxicity and side effects when compared with classic anticancer agents. In addition, considering that drug distribution to the tumor site is low, the use of suitable drug delivery systems (DDS) compatible with the anticancer agents could be explored to achieve better clinical response with lower toxicity, as DDS could be functionalized with ligands that are specifically recognized by cancer cells [49]. Thinking about the fact that HPV infection is localized in the anatomical regions that can be easily reached for topical treatment, the possibility of locally delivering small molecules or natural compounds could be a valid option. Indeed, in low-income countries where women only can reach health facilities a few times in a lifetime, the combination of HR HPV test positivity and treatment in a single visit could be fundamental. A “screen-and-treat” approach allows reducing travel time, minimizing the number of visits, transport, childcare needs, and reducing the cost [61]. In places where is difficult to reach people and a return visit is not an option, self-sampling for HPV screening and mobile treatment for precancer could be applied [62]. Moreover, visual inspection with acetic acid (VIA) can be applied as a triage method for LIC, once it is low-cost and offers the option of treatment immediately or shortly after diagnostic testing. One of the biggest problems of cervical neoplasia is the resistance of tumor cells to chemotherapy and radiotherapy. Thus, the combination of anticancer agents in DDS with conventional chemotherapy or radiotherapy could also be a solution. This represents another line of investigation that needs to be explored in the near future. In terms of CC management, it would be interesting to explore a combinatory approach targeting E6 and E7 oncoproteins. In this case, the aim consists of exploiting molecules able to interact with E6 and E7 proteins, once both have a relevant role on CC progression. From this point forward, the use of in silico methods would be the key to pursue this approach.

Overall, our research group aims to propose a more specific, efficient, and non-toxic/invasive therapeutic approach for cervical cancer management. Thus, in silico approaches, such as those described in the present manuscript, will be used to select promising compounds as E6 potential inhibitors. Additional studies will be conducted with HPV E6 recombinant proteins and the selected compounds to characterize the kinetic magnitude and affinity constants of the compound–protein interaction. Then, the most promising compounds will be applied in in vitro studies with HPV-positive and HPV-negative cell lines to confirm the inhibitor/blocker effects of the E6 oncoprotein. In addition, drug delivery systems can be developed to circumvent a possible high toxicity and low availability of the compounds and, for instance, to combine this approach of E6 inhibitors with gene therapy to supplement p53 content and induce the cancer cell apoptosis.

## 5. Conclusions

The high impact that CC has in developing countries is undeniable. This review has briefly discussed the role of HPV in CC carcinogenesis as well as its different stages and current treatments. Subsequently, the potential of natural and synthetic small molecules in HR HPV E6 protein inhibition, mainly by targeting the E6/E6AP complex or the E6/E6AP/p53 complex, was discussed. The drug development process is a very expensive and time-consuming process until achieving regulatory agency approval. In silico methods represent a viable solution to these current problems by allowing a fast screening and identification of potential drugs and effective predictions. In terms of impact, in silico methodologies can help to increase the speed of acceptance of potential antiviral drugs with ADMET acceptable profiles for CC management. Moreover, in silico methods have been applied in the medical field, representing the therapeutic response of drugs on virtual organs and body systems and predicting patients’ biological responses to the treatment, and this significantly improves outcomes. Computational-based approaches hold a great promise for improving drug development and revolutionizing clinical research by providing a specific treatment for women diagnosed with cervical cancer. Considering the costs of screening and treatment of CC, and knowing that the highest incidence occurs in developing countries, a more cost-effective treatment is needed. Thus, exploring natural compounds with the ability to impair E6-p53 interaction could be a specific and promising strategy for CC management in a more economical way.

## Figures and Tables

**Table 1:** CC stages according to the FIGO system and the recommended treatment.

Stage	Substage	Description	Recommended Treatment
I	IA1	The disease is only found in the cervix	Conization or cone biopsy; simple hysterectomy
	IA2		Conization; simple hysterectomy with pelvic lymphadenectomy; radical hysterectomy
	IB1		Radical hysterectomy or radical trachelectomy with lymphadenectomy
	IB2		
II	IIA1	Outside the cervix, cancer has progressed to the upper vaginal wall or tissue next to the cervix (parametrium), but not to the pelvic sidewall(s)	Radical hysterectomy or radical trachelectomy with lymphadenectomy
	IIA2		Radiotherapy (RT) with or without chemotherapy
	IIB		Cisplatin-based chemotherapy and concurrent radiation (CRT)
III	IIIA	Cancer has advanced to the lower region of the vagina and through the parametrium until the pelvic sidewall(s)	Cisplatin-based chemotherapy and concurrent radiation
	IIIB		
IV	IVA	Cancer has advanced to adjacent organs or distant tissue, such as lungs and distant lymph nodes	Palliative radiotherapy or palliative systemic cytotoxic chemotherapy
	IVB		

## Data Availability

Data sharing not applicable.
